# Antimicrobial Peptide Cec4 Eradicates the Bacteria of Clinical Carbapenem-Resistant *Acinetobacter baumannii* Biofilm

**DOI:** 10.3389/fmicb.2020.01532

**Published:** 2020-08-11

**Authors:** Weiwei Liu, Zhaoying Wu, Chengju Mao, Guo Guo, Zhu Zeng, Ying Fei, Shan Wan, Jian Peng, Jianwei Wu

**Affiliations:** ^1^Immune Cells and Antibody Engineering Research Center of Guizhou Province, Guizhou Medical University, Guiyang, China; ^2^Key Laboratory of Environmental Pollution Monitoring and Disease Control, Ministry of Education, Guizhou Medical University, Guiyang, China; ^3^The Key and Characteristic Laboratory of Modern Pathogen Biology, Basic Medical College, Guizhou Medical University, Guiyang, China; ^4^The Center for Clinical Laboratories, The Affiliated Hospital of Guizhou Medical University, Guiyang, China

**Keywords:** antimicrobial peptide, Cec4, CRAB, biofilm, transcriptome

## Abstract

The drug resistance rate of *Acinetobacter baumannii* increases year on year, and the drugs available for the treatment of carbapenem-resistant *A. baumannii* (CRAB) infection are extremely limited. *A. baumannii*, which forms biofilms, protects itself by secreting substrates such as exopolysaccharides, allowing it to survive under adverse conditions and increasing drug resistance. Antimicrobial peptides are small molecular peptides with broad-spectrum antibacterial activity and immunomodulatory function. Previous studies have shown that the antimicrobial peptide Cec4 has a strong effect on *A. baumannii*, but the antibacterial and biofilm inhibition of this antimicrobial peptide on clinical carbapenem resistance *A. baumannii* is not thoroughly understood. In this study, it was indicated that most of the 200 strains of CRAB were susceptible to Cec4 with a MIC of 4 μg/ml. Cec4 has a strong inhibitory and eradication effect on the CRAB biofilm; the minimum biofilm inhibition concentration (MBIC) was 64–128 μg/ml, and the minimum biofilm eradication concentration (MBEC) was 256–512 μg/ml. It was observed that Cec4 disrupted the structure of the biofilm using scanning electron microscopy (SEM) and confocal laser scanning microscopy (CLSM). A comparative transcriptome analysis of the effects of the antimicrobial peptide Cec4 on CRAB biofilm, identified 185 differentially expressed genes, including membrane proteins, bacterial resistance genes, and pilus-related genes. The results show that multiple metabolic pathways, two-component regulation systems, quorum sensing, and antibiotic synthesis-related pathways in *A. baumannii* biofilms were affected after Cec4 treatment. In conclusion, Cec4 may represent a new choice for the prevention and treatment of clinical infections, and may also provide a theoretical basis for the development of antimicrobial peptide drugs.

## Introduction

*Acinetobacter baumannii* is a non-fermenting Gram-negative bacterium, and infections often occur in patients with poor immunity, especially in patients in intensive care units or in patients undergoing invasive surgery (Handal et al., 2017). It can cause hospital-acquired pneumonia, especially ventilator-associated pneumonia, bacteremia, urinary tract infection, secondary meningitis, etc., with a high mortality rate (Bentancor et al., 2012). At the same time, *A. baumannii* is prone to drug resistance and is even resistant to carbapenem antibiotics, including imipenem and meropenem (Maragakis and Perl, 2008). At present, most antibiotics, except tigecycline and polymyxin, do not affect it (Dafopoulou et al., 2019). Although tigecycline has a broad antibacterial spectrum and good antibacterial activity, the blood concentration is low, only 0.7–0.8 mg/L (Cai and Wang, 2011). Therefore, it remains controversial to use tigecycline to treat blood-related infections (Akalay et al., 2020); moreover, polymyxin has greater renal and neurotoxicity, limiting its use (Spapen et al., 2011). By organism, the highest overall rates of multidrug resistance reported in a study were among *A. baumannii* isolates, for which 44% of isolates collected globally were multidrug-resistant bacteria. Additionally, the treatment options for infections caused by such organisms are limited, which deserves attention (Giammanco et al., 2017). Recently, the World Health Organization (WHO) classified carbapenem-resistant *A. baumannii* (CRAB) as the first in the list of key pathogens for the development of new antibiotics (O’Shea, 2012).

When *A. baumannii* forms biofilms, its resistance increases rapidly (Eze et al., 2018). Biofilms are composed of bacteria that irreversibly adhere to the surface of living or non-living organisms and are surrounded by a secreted matrix of extracellular polysaccharide, protein, and DNA. Once the special structure forms, the bacteria express completely different genes from planktonic bacteria, with significant differences in morphology, physical and chemical properties, and antibiotic susceptibility (Jamal et al., 2018). The ability to form biofilms on abiotic surfaces under adverse conditions makes the biofilm phenotype an important virulence factor for *A. baumannii* infection (Eze et al., 2018). At the same time, it is beneficial for bacteria to survive on nutritionally limited abiotic surfaces and stressful environmental conditions (Alvarez-Fraga et al., 2016). Bacterial biofilms cause at least 65% of human infections, particularly implantable device-related infections and chronic disease infections (Williams and Costerton, 2012). Therefore, there is an urgent need for drugs that effectively treat biofilm-associated infections. At present, it has been reported that some natural product extracts and compounds can treat bacterial biofilms. Water extract of *Galla chinensis* suppressed biofilm and extracellular matrix formation of *Staphylococcus aureus* (Wu et al., 2019). Qin et al. (2014) reported the effects of two natural compounds on methicillin-resistant *Staphylococcus aureus* (MRSA). Ursolic acid can inhibit the formation of biofilms, while resveratrol combined with vancomycin can inhibit pre-formed mature biofilms. Silver nanoparticles do not affect the growth of planktonic *Staphylococcus aureus* but can reduce the production of biofilms at a concentration of 50 μg/ml (Singh et al., 2019). However, in many cases, these anti-biofilm active ingredients are not sufficient enough to completely inhibit or eliminate bacterial biofilms and lack broad-spectrum anti-biofilm efficacy.

Antimicrobial peptides (AMPs) have a broad antibacterial spectrum and a wide range of sources, and have unique antibacterial mechanisms, making them less prone to drug resistance. It is generally believed that AMPs exert their microbicidal activity mainly through targeting the cell membrane by penetration and cell lysis activities (Aisenbrey et al., 2019). The MIC of human-derived cationic peptide LL-37 and its truncated fragments against drug-resistant *A. baumannii* is 16–32 μg/ml, and it inhibits the formation of biofilms (Feng et al., 2013). The peptide IDR-1018 exhibits broad-spectrum anti-biofilm activity against a variety of hospital pathogens, including *Pseudomonas aeruginosa* and *Klebsiella pneumonia* (de la Fuente-Nunez et al., 2014). Therefore, antimicrobial peptides are expected to become new drugs for the treatment of bacterial biofilm and associated infections (Kim et al., 2020; Neshani et al., 2020). Our previous study found that the peptide Cec4 had a minimum inhibitory concentration (MIC) of 4 μg/ml against an *A. baumannii* reference strain (ATCC19606), which is superior to the reported similar cecropin antimicrobial peptides Cec1, cecropin A and fusion peptide CA. (1–8) M (1–18) (Saugar et al., 2002; Batoni et al., 2011; Long et al., 2017). It was confirmed in the previous report that the semi-inhibitory concentration of Cec4 on the formation of standard *A. baumannii* biofilms (to inhibit the formation of biofilm by 50%) is about 4 μg/ml; this peptide is non-hemolytic on human red blood cells at high concentrations (100 × MIC) (Peng et al., 2019). However, whether the antimicrobial peptide Cec4 can inhibit CRAB and its biofilm to the same extent as with the *A. baumannii* (ATCC19606), remains elusive. Therefore, 200 strains of clinical CRAB were collected, and their ability to form biofilms was tested. Furthermore, the susceptibility and biofilm formation of *A. baumannii* isolates to the antimicrobial peptide Cec4 were evaluated, and the molecular mechanism of Cec4 on bacterial biofilm was analyzed by transcriptome analysis. In conclusion, this study is expected to provide new ideas for the treatment of clinical infections and presents a theoretical basis for research and development into new antibiotics.

## Materials and Methods

### Synthesis and Preparation of Peptides

Antimicrobial peptide Cec4 (GWLKKIGKKIERVGQNTRD ATIQAIGVAQQAANVAATLKGK) was synthesized by Gil Biochemical Co., Ltd., Shanghai. Using solid-phase chemical synthesis, the purity (HPLC) > 97% and the mass of the peptide determined by spectrometry. It was dissolved to 10 mg/ml with distilled deionized H_2_O (ddH_2_O) and stored at −80°C for further analysis.

### Bacterial Isolates and Growth Conditions

From October 2017 to December 2018, 200 isolates of CRAB from clinical samples of patients from the affiliated hospital of Guizhou Medical University were collected and duplicate samples from the same patient were excluded. All of them come from sputum, blood, urine and so on. The collection and use of clinically isolated strains were approved by the Institutional review board (IRB) of Guizhou Medical University, China. The studies involving human participants were reviewed and approved by Guizhou Medical University and the affiliation of the ethics committee. The patients provided written informed consent to participate in this study. Minimum inhibitory concentrations (MICs) of these strains to nine antibiotics including amikacin, ertapenem, imipenem, meropenem, ceftazidime, ciprofloxacin, ceftriaxone, levofloxacin, and cefepime were assessed on MicroScan WalkAway 40-SI Analyzer (SIEMENS, Germany), according to the manufacturer’s instructions. Resistance to cefotaxime was assessed using the standard disc diffusion method (Oxoid, Hampshire, United Kingdom). Interpretive breakpoints for susceptible, intermediate, and resistant were consistent with Clinical and Laboratory Standards Institute guidelines (Humphries et al., 2018). In order to ensure the accuracy of bacterial identification, the 16*SrRNA* and *rpoB* genes of all strains were amplified, and PCR products were sequenced and analyzed. *A. baumannii* (ATCC19606) is a biofilm-forming positive strain. *Escherichia coli* ATCC25922 and *Pseudomonas aeruginosa* ATCC27853 were used as quality control bacteria. The above strains are stored in the Pathogen Biology Laboratory of Guizhou Medical University. Strains were grown on Mueller-Hinton Broth (MHB), Typic Soy Broth (TSB) or Luria-Bertani (LB) agar plates and incubated at 37°C.

### Detecting the Minimum Inhibitory Concentration (MIC) Value

According to a previous study (Wiegand et al., 2008), the MIC of Cec4 peptide against 200 strains of CRAB was assessed using the broth microdilution assay in MHB. After adding different concentrations of antimicrobial peptide Cec4, the 96-well plate was placed in a constant temperature incubator at 37°C. After incubation for 24 h, it was observed. The MIC was defined as the lowest drug concentration that can inhibit bacterial growth by visual evaluation.

### Quantitative Biofilm Formation Assay

According to the crystal violet staining method of a previous study (O’Toole, 2011), the 96-well tissue culture plate method was utilized for a quantitative evaluation of biofilm formation by 200 strains of CRAB. *A. baumannii* ATCC19606 was used as the positive control, and TSB medium without bacteria was used as the negative control. Absorbance at 570 nm (OD570) was measured for each well to obtain quantitative data on biofilm formation as described previously (Badmasti et al., 2015). The mean ± standard deviation of the OD value of the negative control was defined as ODc. Based on the OD value, the strains were divided into the following four groups: the OD value of the test strain was compared with ODc, and OD_570_ ≤ ODc was negative for biofilm formation (−); ODc < OD_570_ ≤ 2 × ODc indicates weak biofilm formation (+); 2 × ODc < OD570 ≤ 4 × ODc indicates moderate biofilm formation (++); 4 × ODc < OD570 indicates strong biofilm formation (+++).

### Detection of the Minimum Biofilm Inhibition Concentration (MBIC) and Minimum Biofilm Eradication Concentration (MBEC)

In order to detect the inhibitory effect of Cec4 peptide on the growth of biofilms, a method described in the literature was used with modifications (Abouelhassan et al., 2017). Briefly, 200 μl of bacterial cells (1 × 10^6^ CFU/ml) of 10 strains (CRAB 3, 4, 53, 55, 78, 117, 120, 128, 130, 136) with the strongest biofilm formation ability was inoculated in a polyethylene 96-well plate; TSB culture medium containing no bacteria was used as the blank control, and the plate was incubated at 37°C for 24 h. The culture medium was subsequently removed, and wells were carefully washed with PBS three times to remove planktonic bacteria. And then, 200 μl of TSB culture medium containing Cec4 in serial doubling dilutions was added to each well. TSB medium without an antimicrobial peptide was used as a negative control, and plates were incubated at 37°C for 24 h. If OD600 < 0.1, there was no bacterial growth, and the lowest concentration without bacterial growth at this time point was recorded; this is the MBIC. Then, cells and peptide in the 96-well cell culture plate were washed with PBS, and 200 μl of TSB culture medium was added to each well. The plate was incubated at 37°C for 24 h to re-grow the surviving biofilm bacteria. An OD600 < 0.1 indicated that there was no bacterial growth. The lowest concentration at which no bacterial growth was recorded is the MBEC. In order to evaluate the eradication efficiency of Cec4 on the biofilm, the culture in the wells was removed and washed with PBS to remove non-adherent cells. The biofilm was quantified by the aforementioned method, and calculated using the equation $\left(1-\frac{\text{OD}\,570\,\mathrm{of}\,\mathrm{the}\,\mathrm{test}}{\text{OD}\,570\,\mathrm{of}\,\mathrm{non}-\mathrm{treated}\,\mathrm{control}}\right)\times 100$.

### Scanning Electron Microscopy (SEM)

According to a previous report (Ramalingam and Lee, 2018), the biofilm of CRAB 55 was cultured *in vitro* in a 6-well plate, and a sterile polylysine-treated cover glass and a sterile medical catheter cut at a length of 1 cm were added in advance as a biofilm growth carrier. Samples were processed by gradient dehydration with 20, 50, 70, 90, and 100% ethanol/tert-butanol mixture. The samples were dried in a critical point dryer and placed in a high vacuum evaporator, then sprayed gold with an ion sprayer and observed using a scanning electron microscope (Hitachi S-3400).

### Confocal Laser Scanning Microscope Analysis

Confocal laser scanning microscopy analysis was carried out according to a previous study (Bortolin et al., 2016). A sterile polylysine-treated cover glass was used as the carrier, and the biofilm of CRAB 55 was cultivated according to the method of the previous step. Then, 1 mM SYTO9 and 10 mM propidium iodide (PI) were added and incubated for 15 min in the dark. An Olympus Fluoview FV1000 confocal microscope (Olympus, Markham, ON, Canada) was used to obtain a fluorescence image. The bottom of the biofilm to the surface was scanned layer by layer along the *Z*-axis to record the earliest and last disappearance of fluorescence, and the corresponding biofilm thickness was calculated accordingly; each layer was 1 μm. The resulting stacks of images were quantified using an image processing package (ImageJ, United States) and subsequently rendered into three-dimensional mode using image analysis software (Imaris 7.2.3, Bitplane, Switzerland).

### Motility Assays

As described by a previous study (McQueary and Actis, 2011), twitching plates were made with 10 g/l tryptone, 5 g/l yeast extract and 5 g/l NaCl and 1% Eiken agar, with different concentrations of Cec4 (or none). A single colony of CRAB 55 was picked from a normal LB agar (1.5%, wt/vol) plate and inoculated vertically to the bottom of the plate, so that it could grow at the intersection of the bottom of the agar layer and the culture dish. The plate was incubated at 37°C for 24 h, and motility was evaluated by observing the formation of a transparent halo around the growing colonies and measuring the diameter. Twitching motility assays were conducted on at least three separate occasions. As described in a previous study (Harding et al., 2013), surface motility plates are comprised of 5 g/l tryptone, 2.5 g/l NaCl and 0.35% Eiken agar, with different concentrations of Cec4 or not. The bacteria were cultured to the logarithmic phase, then a 2 μl of aliquot of an overnight culture was stabbed into the surface of the center of the plate, and motility was measured after incubating the plate at 37°C for 24 h. Surface-associated motility assays were conducted on at least three separate occasions.

### Quantitative RT-PCR

According to a previous study (He et al., 2015), biofilm formation of CRAB 55 was conducted as described above, and after the biofilm had been treated with Cec4 or not, it was scraped from the plate with a cell spatula. Total RNA was extracted using Trizol. RNA was quantified and quality was assessed using a NanoDrop spectrophotometer (ND-2000, Thermo Scientific, Loughborough, United Kingdom), and the final RNA concentration was adjusted to 1 ng/μl. cDNA was synthesized in a 20 μl reaction mixture using a PrimeScript RT reagent Kit with gDNA Eraser. According to the SYBR Premix Ex Taq TM Kit (Takara, Dalian, China) protocol, the reactions were run on an ABI7300 real-time PCR system using a 20 μl reaction volume. Gene expression levels were normalized to the abundance of *A. baumannii* 16*S rRNA*. Target genes included *CsuE*, *BfmR*, *BfmS*, *AbaI*, and *Bap*. The primers were designed with reference to the GenBank sequences. The primer sequences are shown in the Supplementary Table S1.

### RNA-Seq

#### RNA Isolation, Library Construction and Sequencing

The four strains CRAB 4, 55, 78, and 117 with the strong biofilm-forming ability and similar characteristics were selected as four biological replicates. The logarithmic growth culture was washed twice in sterile PBS and diluted to 10^6^ cells/ml in TSB. Then, 4 ml of the culture was added to a flat-bottomed 6-well plate (Corning, United States) and incubated at 37°C for 24 h to form a biofilm. Samples were collected as a control group (Control 1, Control 2, Control 3 and Control 4). For the Cec4 treatment group (ABF1, ABF2, ABF3, and ABF4), after forming mature biofilms, non-adherent cells were removed, and TSB culture solution containing 4 × MIC (16 μg/ml) of Cec4 was added. Biofilm samples were collected 24 h later.

The total RNA of these samples was extracted using Trizol reagent (Sigma-Aldrich, United States) according to the previously described method. The quality and quantity were determined by a NanoPhotometer spectrophotometer (IMPLEN, CA, United States) and an Agilent 2100 bioanalyzer (Agilent Technologies, CA, United States). RNA-seq library construction and RNA sequencing were performed by the Novogene Corporation (Beijing, China). Sequence reads were deposited at the National Center for Biotechnology Information under BioProject PRJNA607078 as SAMN14120700, SAMN14120701, SAMN14120702, SAMN14120703, SAMN14120704, SAMN14120705, SAMN14120706, SAMN14120707^1^.

#### Identification of Differentially Expressed Genes and Annotation

Raw reads were generated from the image data and stored as FASTQ format. Raw data were filtered to remove adaptor contaminated and low-quality sequences and to obtain clean reads. Genomic mapping of the filtered sequence was performed using Bowtie2-2.2.3 (Langmead and Salzberg, 2012). The reference genome and gene model annotation file of *A. baumannii* AB030 was downloaded from GenBank (NZ_CP009257.1). HTSeq v0.6.1 was used to count the read numbers mapped to each gene. The expected number of fragments per kilobase of transcript sequence per million base pairs sequenced (FPKM) of each gene was calculated based on the length of the gene and reads count mapped to this gene (Trapnell et al., 2012). Differential expression analysis of two groups (four biological replicates per group) was performed using the DEGSeq R package (??) (Anders and Huber, 2012). The resulting *p*-values were adjusted using the Benjamini and Hochberg approach for controlling the false discovery rate. Genes with a corrected *p*-value < 0.05 and log_2_(Fold change)>1 found by DEGSeq were assigned as differentially expressed. Gene ontology (GO) enrichment analysis of differentially expressed genes was implemented by the GOseq R package. The GO enrichment analysis of differentially expressed genes was achieved by GOseq software, and KOBAS software was used to test the statistical enrichment of differentially expressed genes in KEGG pathways (Kanehisa et al., 2008; Young et al., 2010). Significantly enriched KEGG pathways and GO terms were identified by a *p*-value < 0.05 using Fisher’s exact test and a *p*-value < 0.01 in the hypergeometric distribution, respectively, and adjusted by false discovery rates (FDR) (Rivals et al., 2007; Boca and Leek, 2018).

#### Statistical Analysis

Statistical analysis was performed using GraphPad Prism software version 6.0 (Graph Pad Software, San Diego, CA, United States) and Student’s *t*-test. Data are expressed as mean ± standard deviation (SD). All experiments were performed in triplicate. A *p*-value < 0.05 was considered statistically significant.

## Results

### Cec4 Inhibits Clinical Resistant Bacteria

**FIGURE 1:**
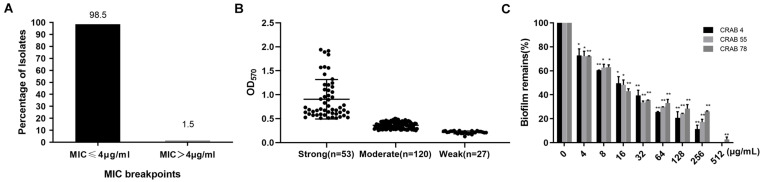
Cec4 inhibits clinical resistant bacteria and biofilms. **(A)** Distribution of MIC value of Cec4 against 200 CRAB clinical isolates (μg/mL). **(B)** Quantification of biofilm formation in 200 CRAB clinical isolates. *A. baumannii* ATCC19606 was used as a positive control. Experiments were performed in triplicate and each bar represents the mean ± standard deviation. **(C)** The effects of Cec4 on mature biofilms of CRAB. The adherent biofilm was stained by crystal violet, and then the dye was extracted with ethanol, measured at a 570-nm absorbance, and presented as percentage of biofilm remains compared to untreated wells (0 μg/mL). All experiments were done in triplicate for statistical significance. *p < 0.05; **p < 0.01.

It was reported that large differences have been shown between *A. baumannii* strains. In previous studies, Cec4 showed outstanding antibacterial activity against *A. baumannii*. Its MIC against standard *A. baumannii*(ATCC19606)was 4 μg/ml. In order to study its antibacterial effect on clinical CRAB, the susceptibility of CRAB to Cec4 was tested. As shown in Figure 1A, 98.5% of the 200 isolates of CRAB were susceptive to the peptide Cec4 with a MIC ≤ 4 μg/ml. Only 1.5% of the strains had MIC > 4 μg/ml, which were 8 and 16 μg/ml, respectively (Supplementary Table S2). Therefore, the peptide Cec4 has great antibacterial effects on the majority of clinical carbapenem resistant bacteria.

### The Biofilm Formation Ability of CRAB Strains

Bacteria in the form of biofilms have increased resistance to antibacterial drugs, external environmental pressures, and the host’s immune system, which has brought great challenges to clinical treatment. Based on crystal violet staining, the ability of 200 isolates of CRAB to form biofilms was studied. As shown in Figure 1B, the OD570 value of the positive control strain ATCC19606 in the experimental plate was 1.191 ± 0.012, and the negative control was 0.111 ± 0.005. The OD570 value of the 200 resistant bacteria was between 0.1 and 1.9 (Supplementary Table S2). All 200 strains of CRAB were able to form biofilms. Among them, 53 strains (26.5%) had strong biofilm formation ability, 120 strains (60%) were moderate and 27 strains (13.5%) were weak.

### Cec4 Inhibits and Eradicates Biofilm of CRAB Strains

After bacteria formed the biofilm, their resistance to drugs was greatly enhanced. Our previous studies have demonstrated that antimicrobial peptide Cec4 at 0.5 μg / mL can inhibit the formation of *A. baumannii* biofilm (Peng et al., 2019). Regarding biofilm formation ability, the 10 strongest strains were selected to evaluate the ability of Cec4 to inhibit and eradicate their biofilm. As shown in Supplementary Table S2, the antimicrobial peptide Cec4 can inhibit the biofilm of the strains with the strongest CRAB biofilm formation ability at 64–128 μg/ml, and can eradicate the biofilm at 256–512 μg/ml. In order to determine the effect of Cec4 on the removal of biofilms, after the formation of mature biofilms, different concentrations of Cec4 were added to the culture for 24 h. The crystal violet staining method was used to measure the absorbance at 570 nm to calculate the clearance rate of different concentrations of the peptide on mature biofilms. The results showed that 1 × MIC (4 μg/ml) could clear more than 20% of mature biofilms, with MBEC_50_ of 16 μg/ml and MBEC_80_ of 128 μg/ml (Figure 1C).

### Cec4 Destroys the Structure of Biofilms

**FIGURE 2:**
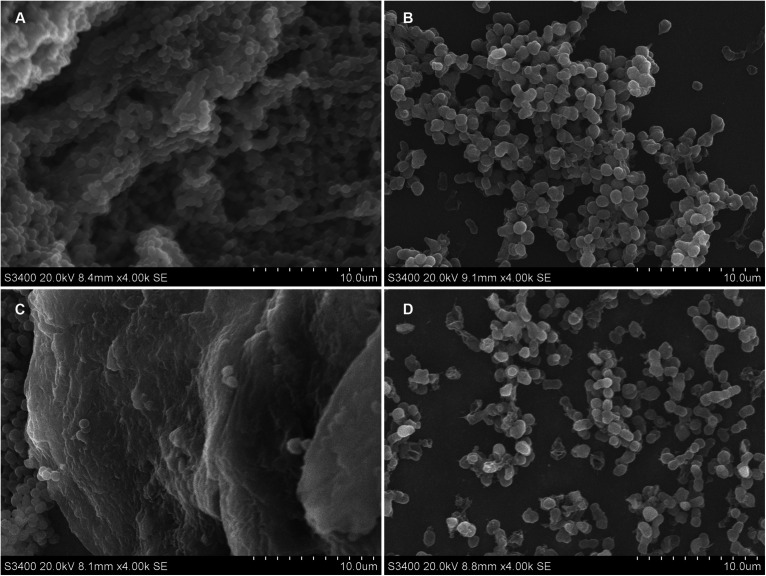
Ultrastructural images of CRAB biofilm on sterile coverslip and catheters after treated with Cec4. **(A)** Coverslip without treatment, **(B)** Coverslip after Cec4 treatment, **(C)** catheter without treatment, and **(D)** catheter after Cec4 treatment. The selected images are the best representation of biofilms on coverslips and catheters.

*Acinetobacter baumannii* colonizes the surface of medical equipment and indwelling medical devices (including urinary catheters) to form biofilms, leading to long-term and recurrent infections in patients (Lin et al., 2019). Coverslips and urinary catheters were used as carriers to evaluate the ability of Cec4 to remove the biofilms formed on them. Clinical CRAB forms biofilm on coverslips and catheters, and the effects of Cec4 on the biofilm are shown in Figure 2. After 24 h in culture, for the untreated group, the *A. baumannii* that adhered to the coverslip mostly was bacilliform; the biofilm was dense and the cell membrane was intact, forming a typical “mushroom cloud” three-dimensional structure. After treatment with Cec4 at a concentration of 1 × MIC (4 μg/ml), the biofilm structure was destroyed, and the cells were loosely distributed, and some cell surfaces appeared to be attacked. *A. baumannii* adhered to the catheter was mostly spherical and formed a thick biofilm, covered with extracellular substrates. After treatment with Cec4 (4 μg/ml), the biofilm structure was destroyed. Moreover, small vesicles appeared on the surface of the cell membrane, and even the collapse and disintegration of cells were observed.

**FIGURE 3:**
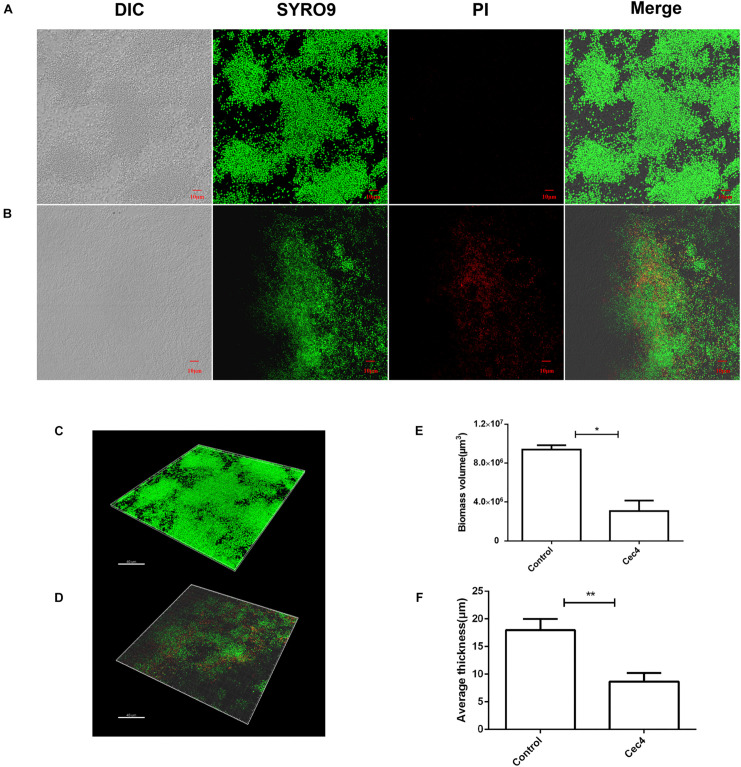
The CRAB biofilm was removed effectively with Cec4. Confocal microscopy of CRAB biofilm without treatment **(A)** and after treatment with Cec4 **(B)**. The three-dimensional reconstruction of CRAB biofilm without treatment **(C)** and after treatment with Cec4 **(D)**. The total biomass volume **(E)** and thickness **(F)** of biofilm were calculated based on the fluorescence intensity. The results were averaged from three randomly selected positions of each sample. Data are expressed as mean ± standard deviation (SD). *p < 0.05; **p < 0.01.

To better understand the destructive effect of Cec4 on the biofilm, the fluorescent dyes SYTO^®^9 and propidium iodide were used to characterize bacteria in different states. Under the laser confocal scanning microscope, a large number of biofilm bacteria were aggregated into the control group, mainly living bacteria with green fluorescence (Figure 3). The total biomass was about 9 × 10^6^ μm^3^ and the average biofilm thickness was > 16 μm. The number of dead bacteria increased gradually after treated with Cec4 (4 μg/ml) for 2 h. It was found that many bacteria in the visual field emitted red fluorescence. The total biomass was about 4 × 10^6^ μm^3^, and the thickness of the biofilm was reduced to 7 μm. Therefore, the CLSM results are consistent with the SEM observations, showing that Cec4 has a strong damaging effect on CRAB strain biofilm.

### Cec4 Decreases Twitching Motility and Surface-Associated Motility in CRAB

**FIGURE 4:**
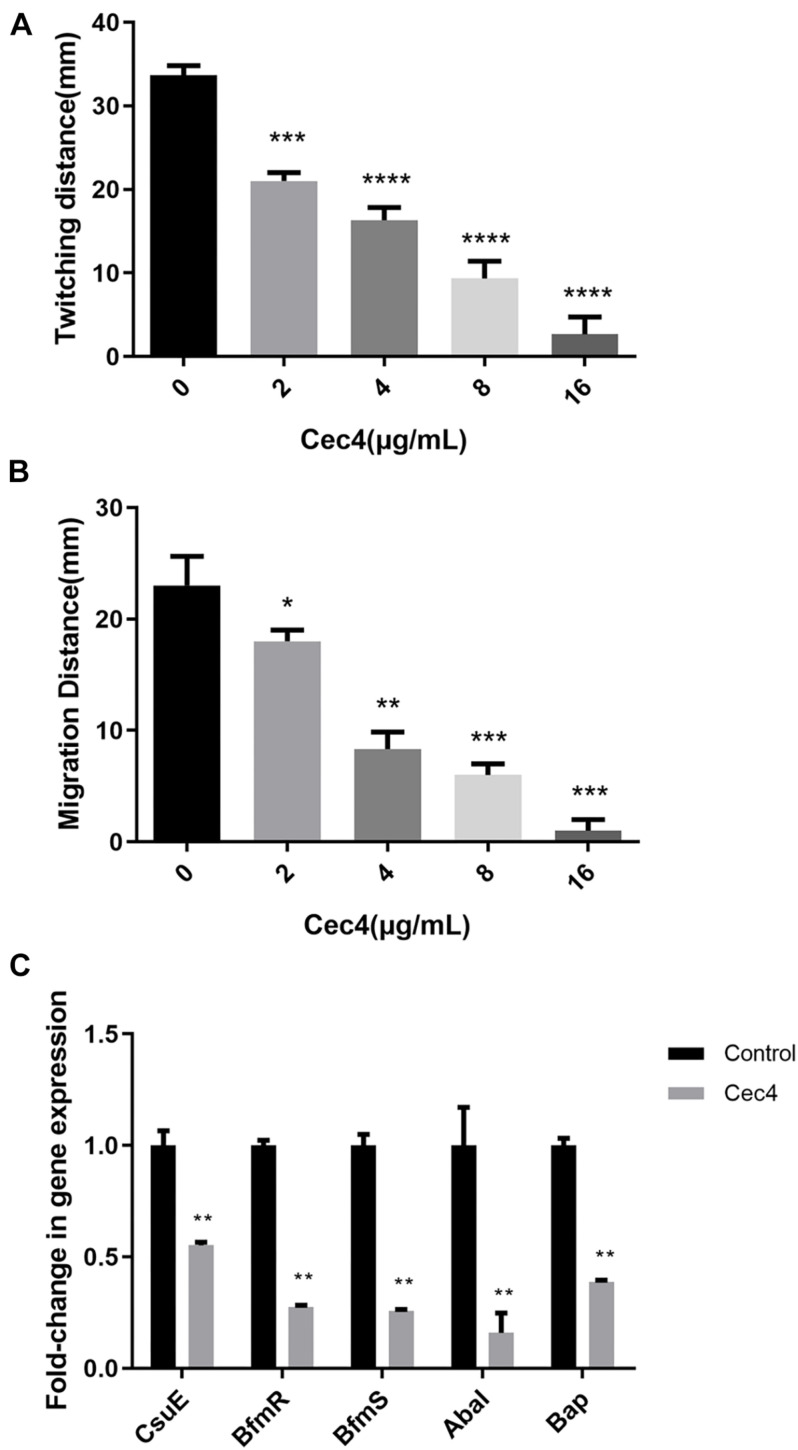
The motility of CRAB and biofilm involved genes were affected after treated with Cec4. Twitching motility **(A)** and surface-associated motility **(B)** of CRAB were quantitated from three separate experiments at 37°C. Error bars represent standard deviation of the mean. *p < 0.05; **p < 0.01; ***p < 0.001; ****p < 0.0001. **(C)** Effect of Cec4 on the expression levels of biofilm involved genes in CRAB strains. Error bars represent the standard deviations. **p < 0.01.

*Acinetobacter baumannii* adheres to the surface of the object through pili, which is the initial stage of biofilm formation. Twitching motility is a unique form of movement mediated by type IV pili. *A. baumannii* relies on the contraction and extension of type IV pili on the surface of the Petri dish, with the inoculation point as the center, and spreads around and forms interstitial colony expansion halo. We observed that, after incubation at 37°C overnight, two types of the colony grew on the Petri plate. There were colonies on the surface of the agar around the inoculation point (top colony) and a visible halo of bacteria that had twitched across the plate between the bottom of the agar and the plate (interstitial colony) (Supplementary Figure S1). Adding different concentrations of Cec4 decreased twitching motility. As the concentration of Cec4 increased, the twitching motility of bacteria decreased (Figure 4A). *A. baumannii* can also move on the surface of a semi-solid medium, forming round colonies at the inoculation site of the moving plate (Supplementary Figure S2). The average colony diameter of the group with Cec4 was lower than that of the control group, which was consistent with the experimental results on twitching motility (Figure 4B).

### Cec4 Alters the Expression of Biofilm-Related Genes

Many studies have shown that several genes of *A. baumannii* are involved in the formation of biofilms and adhesion to abiotic surfaces. In order to understand the effect of Cec4 on these genes, the RNA of biofilm bacteria under different conditions was extracted. Next, the effect of Cec4 on the expression of *CsuE*, *BfmR*, *BfmS*, *AbaI*, and *Bap* related to biofilm formation was evaluated using qRT-PCR (Figure 4C). The results show that, compared with the control group, the pilus-related gene *CsuE* was down-regulated 1.8-fold; the two-component regulatory system genes *BfmR* and *BfmS* were down-regulated 3.6-fold and 3.9-fold, respectively; the quorum-sensing regulatory gene *AbaI* was down-regulated 6.3-fold, and the biofilm-related gene *Bap* was down-regulated 2.6-fold. These results suggest that Cec4 inhibited the expression of these genes involved in biofilm formation.

### Transcriptional Stress Response of CRAB Biofilm to Cec4

**FIGURE 5:**
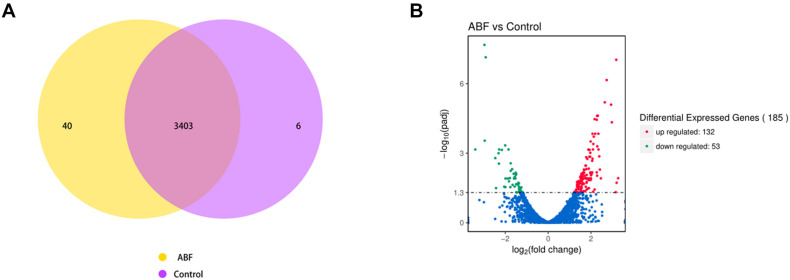
**(A)** Venn diagram analysis of gene expression. The number in each circle represents the total number of genes that are expressed in each sample, and the overlapping part of circles indicates that the gene is co-expressed in both samples. **(B)** The volcano plots of the differentially expressed genes. Significantly differentially expressed genes were treated with red dots (up-regulated) or green dots (down-regulated). The abscissa represents fold change, and the ordinate represents statistical significance.

The RNA sequencing results revealed the differences in gene expression between ABF and control. There were 3403 genes co-expressed in these two samples; 40 genes were specificity expressed in ABF, and 6 genes were specificity expressed in control (Figure 5A). The volcano plots of the differentially expressed genes (DEGs) show that 185 genes were differentially expressed after Cec4 treatment, including 132 genes that were up-regulated (red) and 53 down-regulated genes (green) (Figure 5B).

### Analysis of Differential Gene Expression

Transcriptome analysis revealed that the differential genes mainly included membrane protein-related genes, drug-resistant genes and pilus-related genes (Table 1). The expression of the amino acid ABC transporter permease, ATP-binding proteins (IX87_RS02655, IX87_RS02660, and artP) in the ABC transport system was down-regulated 1.68-fold, 1.61-fold, and 1.66-fold; the MFS transporter (IX87_RS20020) was down-regulated 2.96-fold, and the EamA family transporter (IX87_RS12100) expression was down-regulated 2.29-fold (Table 1). The expression of MBL fold metallo-hydrolase (IX87_RS19070) was down-regulated about 3-fold, ADC family cephalosporin-hydrolyzing class C beta-lactamase (IX87_RS08365) was down-regulated 1.27-fold; adeC/adeK/oprM family multidrug efflux complex outer membrane factor (IX87_RS16845) expression was up-regulated 1.37-fold (Table 1). The protein CsuA (IX87_RS06910) was down-regulated 1.51-fold, while the expression of pili assembly chaperone (IX87_RS02530) and type 4 fimbria biogenesis proteins FimT (IX87_RS10830) was up-regulated 1.8 and 1.42-fold (Table 1).

**TABLE 1:** The genes up-regulated or down-regulated response to Cec4.

**Gene name**	**Description**	**log ^2^ Fold_change**	**Corrected *p*-value**
**Membrane protein**			
IX87_RS20020	MFS transporter	–2.96	2.87 × 10^–4^
IX87_RS02655	amino acid ABC transporter permease	–1.68	4.86 × 10^–3^
artP	amino acid ABC transporter ATP-binding protein	–1.66	2.30 × 10^–2^
IX87_RS02660	amino acid ABC transporter permease	–1.61	7.77 × 10^–3^
IX87_RS12100	EamA family transporter	–2.29	6.85 × 10^–4^
**Bacterial resistance**			
IX87_RS19070	MBL fold metallo-hydrolase	–2.97	2.11 × 10^–8^
IX87_RS08365	ADC family cephalosporin-hydrolyzing class C beta-lactamase	–1.27	4.86 × 10^–2^
IX87_RS16845	adeC/adeK/oprM family multidrug efflux complex outer membrane factor	1.37	2.90 × 10^–2^
**Pili**			
IX87_RS06910	protein CsuA	–1.51	8.64 × 10^–3^
IX87_RS02530	pili assembly chaperone	1.8	1.23 × 10^–2^
IX87_RS10830	type 4 fimbrial biogenesis protein FimT	1.42	3.63 × 10^–2^

### Enrichment Analysis of GO and KEGG Pathways

**FIGURE 6:**
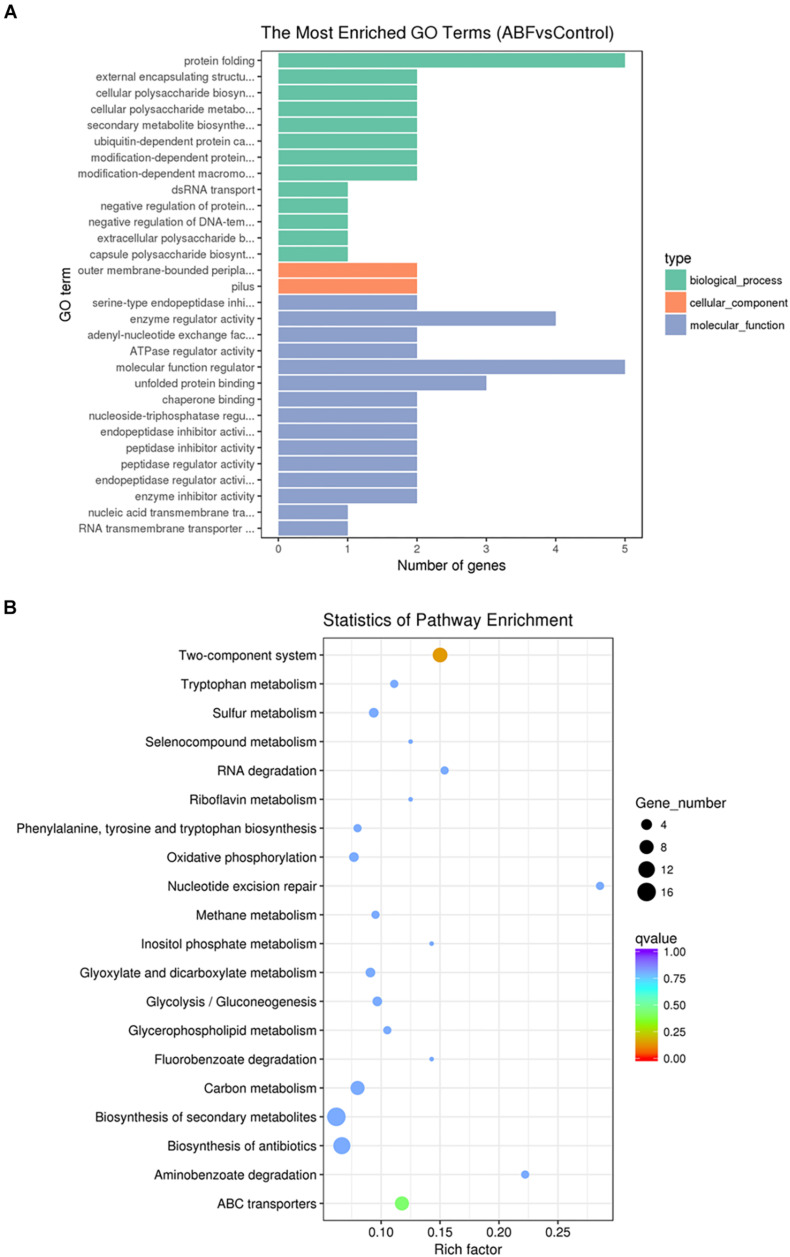
**(A)** Gene Ontology (GO) analysis of differentially expressed genes. **(B)** Statistical enrichment of differentially expressed genes in KEGG pathways.

To further understand the function of the DEGs underlying the effect of low concentrations of Cec4 on CRAB biofilms, GO enrichment analysis was performed with the DEGs (Figure 6A). Based on sequence homology, DEGs were assigned to one or more GO terms and categorized into three main categories of GO function (biological process, molecular function, and cellular component). It is noteworthy that in the category of biological process, protein folding, extracellular polysaccharide biosynthetic process, cellular polysaccharide metabolic process, secondary metabolite biosynthetic process, protein catabolic process, dsRNA transport and regulation of DNA-templated transcription are significantly enriched. Outer membrane-bounded periplasmic space and pilus are significantly enriched in the cellular component category. Enzyme activity and nucleic acid transmembrane transporter activity are enriched in the molecular function category.

By enrichment analysis, the main KEGG metabolic pathways involved in DEGs are carbon metabolism, oxidative phosphorylation, the two-component system, ABC transporters, nucleotide excision repair, biosynthesis of antibiotics, quorum sensing, microbial metabolism in diverse environments, and β-lactam resistance (Figure 6B). It is indicated that Cec4 affected the energy metabolism of biofilm bacteria and the bacteria two-component system. By interfering with the expression of type IV pili assembly protein in the two-component system, bacterial motility function was affected. In addition, the β-lactam antibiotics are the most widely used group of antibiotics, which exert their effect by interfering with the structural cross-linking of peptidoglycans in bacterial cell walls. Bacterial resistance to β-lactam antibiotics can be achieved by producing inactivating enzymes called β-lactamases, altering the β-lactam targets of penicillin-binding proteins (PBPs), and so on. After Cec4 treatment, genes in the β-lactam resistance pathway were down-regulated. More importantly, quorum sensing (QS) allows bacteria to share information about cell density and adjust gene expression accordingly, and controls including virulence, motility, sporulation and biofilm formation. Therefore, the transcriptome results indicate that Cec4 mainly affected the expression of energy metabolism and quorum sensing signaling pathway genes.

## Discussion

It has been reported that after bacteria form a biofilm, the MIC value for antibiotics can be increased by 1000 times or more (Alhede et al., 2011). The antimicrobial peptide can down-regulate quorum sensing, prevent the initial adhesion of bacteria to the surface, target bacteria before they form a biofilm, kill bacteria embedded in biofilms, or destroy mature biofilms to achieve the effect of inhibiting or eradicating biofilms (Batoni et al., 2016). Previous experiments have shown that Cec4 has the best antibacterial effect on *A. baumannii*, and it has very low hemolysis to human red blood cells even in high concentrations (600 μg/mL) (Peng et al., 2019). Our experiment results also indicated that Cec4 had little cytotoxic effect on the two human cell lines HepG2 and Hela (data not shown). In this study, 200 isolates of CRAB from different clinical patients were collected to detect their biofilm formation ability and to evaluate the inhibitory effect of Cec4 on biofilms. Screening at a concentration of 4 μg/ml revealed that 98.5% of the strains were susceptive to Cec4. Thus, Cec4 has a strong inhibitory activity against standard *A. baumannii* and can also inhibit clinical CRAB. The crystal violet staining method showed that the collected clinical CRAB could form biofilms, and 26.5% of the strains could strongly form a biofilm. Cec4 eradicated more than 20% of mature biofilms at a concentration of 4 μg/ml, and the MBEC_50_ was 16 μg/ml. When treated with Cec4 with a concentration of 256–512 μg/ml, the biofilm was completely eradicated. The SEM results indicated that the biofilm formed on the medical silicone catheter was thick, with extracellular polysaccharides and other substrates that completely covered the cells. After Cec4 was treated for 2 h, the biofilm was easily detached, and the cells were damaged or collapsed. The CLSM results further quantified the thickness and volume of the biofilms. The results show that, after 24 h, the biofilm that formed on the coverslips was thick and dense, and mainly composed of live bacteria with a thickness of 10–20 μm. After treatment with Cec4, the number of dead bacteria gradually increased, and the biofilm became significantly thinner. Therefore, these results indicate that Cec4 has a good eradication ability on biofilm forming of clinical CRAB.

It has been reported that *A. baumannii* adheres to the surface of living or non-living organisms by pili participating in the initial stage of biofilm formation, and thereby forming a biofilm. The results show that Cec4 decreased pili-mediated motility in a concentration dependent manner. Under the action of Cec4 with a sub-inhibitory concentration (2 μg/ml), motility was also suppressed, and no transparent halo was formed. It has been reported that peptide 1037 inhibited the swimming of *P. aeruginosa* PA14, but stimulated twitching motility (de la Fuente-Nunez et al., 2012). The expression of the CsuA/BABCDE chaperone complex is necessary for the assembly and production of pili associated with abiotic surface adhesion, while the Csu operon is controlled by a two-component system consisting of a BfmS-encoded sensor kinase and a BfmR-encoded response regulator. Insertion inactivation of BfmR results in the loss of expression of the CsuA/BABCDE operon, and impaired pilus production and biofilm formation (Tomaras et al., 2008). The qRT-PCR results show that Cec4 reduced the expression of *CsuE*, *BfmR* and *BfmS* genes. Clinical isolates of *A. baumannii* M2 can produce N-acyl-homoserine lactone (3-OH-C12-HSL), a product of the *abaI* autoinducer synthetase gene, which is very important for the formation of complete biofilms on abiotic surfaces (Niu et al., 2008). Our research shows that Cec4 significantly reduced the expression of *AbaI*. Therefore, we speculate that Cec4 may affect quorum sensing in bacteria. In addition, *A. baumannii* encodes for a protein associated with the biofilm, Bap. *A. baumannii* strain 307–0294, mutations in large outer membrane proteins that are highly similar to *Staphylococcus* biofilm-associated protein (Bap) were lost, resulting in a reduction in the volume and thickness of the biofilm formed by the strain (Loehfelm et al., 2008). Cec4 reduced the expression of the *Bap* gene, which was consistent with its effect on the biofilm. In conclusion, Cec4 affects the expression of genes involved in the formation of *A. baumannii* biofilms, such as quorum-sensing and motility genes.

Through the neutralization or decomposition of LPS and the interference of gene expression, abnormal regulation of the genes for biofilm survival, thus inhibiting the formation of biofilm (Li et al., 2004). It has been reported that peptide 1018 can inhibit bacterial stress response via the (p)ppGpp signaling molecule (Reffuveille et al., 2014); however, there are few reports on the mechanism of inhibition and eradication of biofilm by antimicrobial peptides. To further investigate the mechanism of action of Cec4, we used RNA-Seq to study the transcriptomic profile of CRAB biofilms treated with Cec4. The RNA-Seq results show that gene expression of CRAB biofilms was extensively altered after Cec4 treatment. Compared with the control group, membrane proteins, bacterial resistance and pilus-related genes were differentially expressed. Iron ion as an important signal regulator mediates the expression of adhesion, and then affects the formation of biofilms (Gentile et al., 2014). Our results show that bacterial ferritin was down-regulated, iron ion uptake and storage-related gene expression were increased. Besides, drug efflux-related genes and ABC transporter-related genes expressions in iron-containing cells were up-regulated. GO terms were abundant in biological process categories, indicating that bacterial biological processes were significantly affected, including the synthesis and metabolism of polysaccharides and proteins. In addition, KEGG analysis showed that multiple metabolic pathways, two-component regulation systems, quorum sensing and antibiotic synthesis-related pathways in *A. baumannii* biofilms were affected after Cec4 treatment. Most importantly, the two-component signal transduction system regulates ABC transporters and type IV pili assembly proteins and participates in the growth and formation of biofilm bacteria (Xue et al., 2020). Whiteley et al. (2017) mentioned that quorum sensing is involved in the formation of biofilms. Cec4 may inhibit the growth of biofilms by affecting the quorum sensing signaling pathway. Therefore, the results show that Cec4 regulates the CRAB biofilm through multiple targets, and further experiments such as gene knockout verification are required to determine the key anti-biofilm mechanisms.

In summary, this study showed that Cec4 has good antibacterial activity against planktonic clinical CRAB and its biofilm. However, the biofilm formation of clinical strains has great differences. It is reported that resistant strains achieve high levels of biofilm-specific resistance despite producing weak biofilms (Qi et al., 2016). So, the epidemiological analysis of the 200 clinic CRAB strains is very important in understanding their genetic relationship. For example, it was shown that, of the cases of *A. baumannii* acquisition, at least 17% were cases of patient-to-patient transmission in the Intensive Care Unit (Harris et al., 2019). Furthermore, deeper explorations of epidemiologic studies, such as bacterial molecular typing, drug resistance, and virulence factors detecting clinical strains, would improve our understanding of their relationship. In conclusion, these results provide a new strategy for the treatment of clinical biofilm-related infections, and also lay the foundation for the development of antimicrobial peptides as new antibacterial drugs.

## Data Availability Statement

The sequencing data in the article have been deposited at the National Center for Biotechnology Information under BioProject PRJNA607078 as SAMN14120700, SAMN14120701, SAMN14120702, SAMN14120703, SAMN14120704, SAMN1412 0705, SAMN14120706, and SAMN14120707 (https://www.ncbi.nlm.nih.gov/sra/PRJNA607078).

## Author Contributions

ZW and WL conceived and designed the experiments. ZW, WL, and CM performed the experiments. WL, ZW, ZZ, and GG analyzed the data. YF, SW, and ZZ contributed materials and analysis tools. JP and JW wrote the manuscript. All authors analyzed the data and contributed to the manuscript, gave final approval of the version to be published, and agreed to be accountable for all aspects of the work.

## Conflict of Interest

The authors declare that the research was conducted in the absence of any commercial or financial relationships that could be construed as a potential conflict of interest.

## Footnotes

1 https://www.ncbi.nlm.nih.gov/sra/PRJNA607078

## Supplementary Material

FIGURE S1 Images of twitching motility after treated with Cec4. From left to right **(A–E)**, the concentration of Cec4 is 0, 2, 4, 8, 16 μg/ml.

FIGURE S2 Images of surface-associated motility after treated with Cec4. From left to right **(A–E)**, the concentration of Cec4 is 0, 2, 4, 8, 16 μg/ml.

TABLE S1 Oligonucleotide primers used in this study.

TABLE S2 The MICs, MBIC, MBEC, sample source and drug susceptibility of 200 CRAB strains.
