# Supplementary figures and images for: Antimicrobial Peptide Cec4 Eradicates the Bacteria of Clinical Carbapenem-Resistant Acinetobacter baumannii Biofilm
Source: Front Microbiol. 2020 Aug 11;11:1532. doi: 10.3389/fmicb.2020.01532 (PMC7431629; doi:10.3389/fmicb.2020.01532)

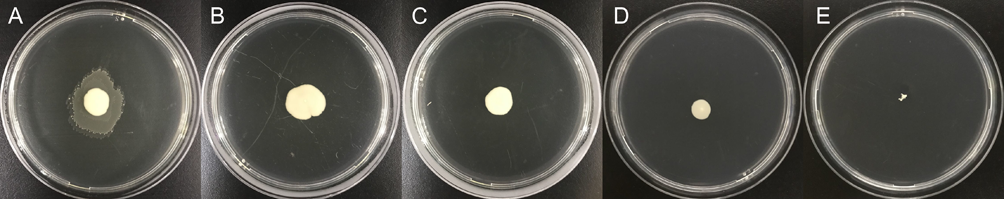

Supplement: FIGURE S1 — Images of twitching motility after treated with Cec4. From left to right (A–E), the concentration of Cec4 is 0, 2, 4, 8, 16 μg/ml. [file Image_1.tif]

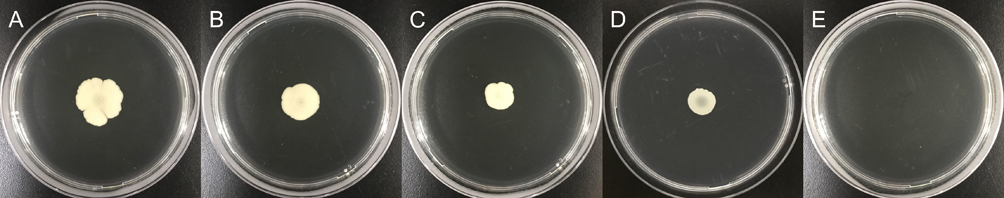

Supplement: FIGURE S2 — Images of surface-associated motility after treated with Cec4. From left to right (A–E), the concentration of Cec4 is 0, 2, 4, 8, 16 μg/ml. [file Image_2.tif]
