# Supplementary material for: Antimicrobial Peptide Cec4 Eradicates the Bacteria of Clinical Carbapenem-Resistant Acinetobacter baumannii Biofilm
Source: Front Microbiol. 2020 Aug 11;11:1532. doi: 10.3389/fmicb.2020.01532 (PMC7431629; doi:10.3389/fmicb.2020.01532)
Supplement: TABLE S1 — Oligonucleotide primers used in this study. [file Table_1.docx]

**Table S1** **Oligonucleotide primers used in this study**

| **Gene** | **Primer sequences(5'➔3')** | **Annealing**  **temperature(°C)** | **Product**  **size (bp)** | **References** |
| --- | --- | --- | --- | --- |
| *csuE* | TTGTGGGAATCGGGGTGTTCTTTG | 60 | 103 | This study |
|  | GAGAGTGAACCAAGCGAGTCTGC |  |  |  |
| *bfmR* | GTCGTGAAGTTCGCCCACACTATC | 60 | 94 | This study |
|  | GCACCCATTTCCAGACCAAGTACC |  |  |  |
| *bfmS* | GCAGAGCGTCGTTACCTTCACC | 60 | 92 | This study |
|  | ATACCGCCCGTAATCCGAACTTTG |  |  |  |
| *abaI* | AGACTACTACCCACCACACAACCC | 60 | 148 | This study |
|  | GACTGCTAGAGGAAGGCGGTTTTG |  |  |  |
| *bap* | AACGACAGCACACCAGCACTTAC | 60 | 106 | This study |
|  | TGCCGTCACCATTGTTCACTGC |  |  |  |
| 16*S rRNA* | TACACACCGCCCGTCACACC | 60 | 119 | This study |
|  | CGGCTACCTTGTTACGACTTCACC |  |  |  |

Primers were designed by Sangon Biotech (Shanghai)
